# Gender perspectives on views and preferences of older people on exercise to prevent falls: a systematic mixed studies review

**DOI:** 10.1186/s12877-017-0451-2

**Published:** 2017-02-17

**Authors:** Marlene Sandlund, Dawn A. Skelton, Petra Pohl, Christina Ahlgren, Anita Melander-Wikman, Lillemor Lundin-Olsson

**Affiliations:** 1 Department of Community Medicine and Rehabilitation, Physiotherapy, Umeå University, Umeå, Sweden; 2 School of Health and Life Sciences, Glasgow Caledonian University, Glasgow, UK; 3 Department of Rehabilitation and Department of Medical and Health Sciences, Linköping University, Linköping, Sweden; 4 Department of Health Sciences, Division of Health and Rehabilitation, Luleå University of Technology, Luleå, Sweden

**Keywords:** Accidental falls, Adherence, Aged, Exercise, Gender identity

## Abstract

**Background:**

To offer fall prevention exercise programs that attract older people of both sexes there is a need to understand both women’s and men’s views and preferences regarding these programs. This paper aims to systematically review the literature to explore any underlying gender perspectives or gender interpretations on older people’s views or preferences regarding uptake and adherence to exercise to prevent falls.

**Methods:**

A review of the literature was carried out using a convergent qualitative design based on systematic searches of seven electronic databases (PubMed, CINAHL, Amed, PsycINFO, Scopus, PEDro, and OTseeker). Two investigators identified eligible studies. Each included article was read by at least two authors independently to extract data into tables. Views and preferences reported were coded and summarized in themes of facilitators and barriers using a thematic analysis approach.

**Results:**

Nine hundred and nine unique studies were identified. Twenty five studies met the criteria for inclusion. Only five of these contained a gender analysis of men’s and women’s views on fall prevention exercises. The results suggests that both women and men see women as more receptive to and in more need of fall prevention messages. The synthesis from all 25 studies identified six themes illustrating facilitators and six themes describing barriers for older people either starting or adhering to fall prevention exercise. The facilitators were: support from professionals or family; social interaction; perceived benefits; a supportive exercise context; feelings of commitment; and having fun. Barriers were: practical issues; concerns about exercise; unawareness; reduced health status; lack of support; and lack of interest. Considerably more women than men were included in the studies.

**Conclusion:**

Although there is plenty of information on the facilitators and barriers to falls prevention exercise in older people, there is a distinct lack of studies investigating differences or similarities in older women’s and men’s views regarding fall prevention exercise. In order to ensure that fall prevention exercise is appealing to both sexes and that the inclusion of both men and women are encouraged, more research is needed to find out whether gender differences exists and whether practitioners need to offer a range of opportunities and support strategies to attract both women and men to falls prevention exercise.

**Electronic supplementary material:**

The online version of this article (doi:10.1186/s12877-017-0451-2) contains supplementary material, which is available to authorized users.

## Background

Falls present the most common cause of injury in old age. At least one third of people aged 65-years and above fall every year, half of them more than once [1], and the incidence increases with advancing age [2]. Women are more prone to falling compared to men and sustain more fall related injuries [3]. The annual rates of non-fatal injuries due to falls for women have been reported to be 48.4% higher than the rates for men [4]. However, a recent study has shown that when the values for comorbidities, lean and fat body mass, and balance were similar between men and women, men actually demonstrated a higher probability of falling [5]. Indeed, men in all age groups are more likely than women to suffer from a fatal fall injury [4]. It is, therefore, important to consider men in fall prevention research and interventions.

According to recent systematic reviews and meta-analyses on interventions to reduce falls, exercise programs that focus on balance combined with muscle strength in the lower limbs are effective interventions to address risk and rate of falling [6, 7]. Exercise programs delivered at home with support from health professionals are cost effective, at least in adults 80 years and older, and cost neutral in those younger [8]. However, both group and home based fall prevention exercise programs are effective as long as they are performed for an effective length of time, regularly enough, and include adequate strength and balance progression [7, 9].

Despite consistent evidence that strength and balance training is effective in reducing falls and fall related injuries across a range of ages and settings, participant uptake is often poor. In a recent systematic review of older people’s participation in and engagement with fall prevention interventions in community settings, only 64.2% accepted the invitation to join the exercise intervention and 19.4% then dropped out of the intervention when they learned what the intervention entailed. However, once they started the intervention there was a 90% retention to the end of the intervention [10]. Average adherence to group based exercise programs have been estimated to around 75% [11], and for home based long term training, adherence rates lower than 50% have been reported [12]. Previous reviews investigating views and preferences of older people for general fall prevention programs have found that ‘autonomy supportive’ programs, perceived as relevant and life-enhancing, facilitated participation. Such programs included education, involvement in decision-making, individually tailored interventions and social support [13, 14].

Research has tended to focus on women in fall prevention interventions. Between 70 and 77% of participants in reviews of intervention studies were women [6, 9]. Men are less likely than women to report falls, seek medical care, and/or discuss falls and fall prevention with a healthcare provider [15]. In order to offer fall prevention exercise programs that attract older people of both sexes there is a need to understand both women’s and men’s views and preferences regarding these programs. With the exception of walking, which is the most common type of leisure time physical activity for both women and men in most cultures, women tend to prefer different types of physical activity than men [16]. Different exercise programs may have different ‘meanings’ to men and women and the way in which we market these opportunities or how they are run may have differing effects in terms of uptake and adherence for them. Therefore, careful analysis of womens’ and mens’ views and preferences to the delivery of falls prevention exercise is crucial.

No previous reviews have explicitly considered similarities or differences between men and women in their views or preferences. Therefore, the aim of this study was to review the literature and explore underlying gender perspectives within older people’s views or preferences regarding uptake and adherence to exercise to prevent falls.

## Method

Although this review was interested in gender perspectives, a pilot scoping review prior to this study suggested very little literature with gender views or preferences specifically. Therefore it was decided not to apply an inclusion criteria based on gender, but instead review all current literature on the topic and extract any gender information reported within the wider literature for this review. The research question was “What are the views and preferences of older adults in exercise to prevent falls and are there any differences between women and men?”

### Criteria for considering studies for this review

#### Types of studies

Both quantitative, qualitative and mixed methods articles were included in this review to ensure this review was as inclusive as possible. The key feature of this review was to get a wide scope of the literature with breadth and depth. Previous reviews have often rated the quality of studies so the present review, which updates previous reviews but whose aim is to consider the gender perspectives, did not entail the appraisal and exclusion of articles based on the quality of research methodology [17].

#### Participant criteria

Trials were included if they specified an inclusion criterion of 60 years of age or over. Trials that include younger participants were included if the mean age minus one standard deviation was more than 60 years. Participants could be community dwelling or living in residential settings but not if they were currently in hospital settings.

#### Inclusion criteria

- majority of participants being aged ≥ 60 years (see above)
- living in the community or in care home settings, with any medical condition[s]
- presented the views and preferences on fall prevention exercise programs by the older people

#### Exclusion criteria

- studies not reporting views on exercise to prevent falls
- studies not specific to fall prevention
- studies which only reported adherence to a program or program components, not reasons for non-adherence
- studies limited to perspectives of significant others or personnel
- patients within a hospital ward setting
- studies not presented in English

### Literature search

The electronic databases PubMed, CINAHL, Amed, PsycINFO, Scopus, PEDro, and OTseeker were searched for applicable studies, up to February 2016. A professional librarian was consulted to plan the search strategy. The search terms were free-text and ‘medical subject heading’ (MeSH) terms combined with appropriate Boolean operators. An additional file explains the search strategy in more detail (see Additional file 1). Limits were set to peer reviewed articles written in English, concerning human subjects. In addition the reference lists of all included papers and identified reviews [13, 14, 18, 19] were screened for further articles.

### Study selection process and data extraction

Search returned titles were reviewed for inclusion by two authors (MS/LLO). In all cases of uncertainty the abstract or the full text article was read. Discrepancies were discussed and resolved between the two reviewers, where necessary, involving one of the other authors. Data extraction and analysis of the included articles was performed by two pairs of authors (all authors read a selection of articles) independently. Data was extracted into tables summarizing: aim; methods; number and characteristics of participants; participant’s views and preferences on fall prevention exercise programs. Notes were made on any gender perspectives found or reported. Profession and gender of the authors was extracted by the first author. The final data extraction tables were checked by all authors. Studies were not ranked on quality.

### Data synthesis

Since studies of all designs were included, the standard systematic review steps for mixed studies reviews with a convergent qualitative design was used to synthesize the results [20]. The views and preferences reported in the articles were coded and categorized by the first author into *facilitators* and *barriers* to falls prevention exercise. Within these two categories the codes were analyzed and summarized into themes using a thematic analysis approach [21]. The first author made the first analysis into themes and these were later presented and discussed within the author group until consensus was reached. In addition, all quotes related to gender differences in the participant’s views and preferences were identified. However, due to potential bias in the selection of quotes made by the original studies authors, and a limited number of quotes found, no formal analyses of these quotes was performed.

## Results

### Characteristics of the included studies

**Fig. 1:**
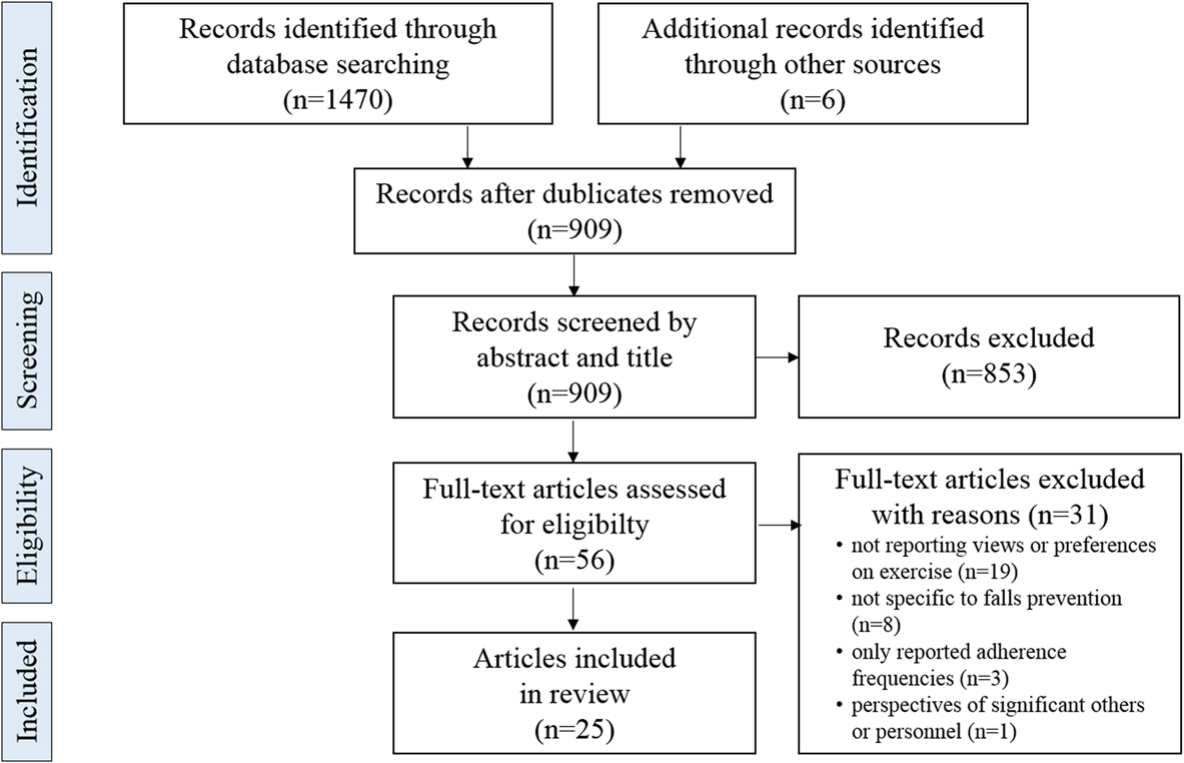
Details on the process of including papers for this mixed studies review

The literature search and inclusion of articles are presented, as recommended, following the PRISMA guidelines [22]. A total of 1476 articles were identified in the search. After removing duplicates, 909 abstracts were screened, 56 met the inclusion criteria. After full text screening, 25 of the retrieved papers were considered appropriate to include in this review based on the inclusion and exclusion criteria [23–47] (Fig. 1).

The aim, study design, and number of participants of the included studies are presented in Tables 1 (quantitative and mixed) and 2 (qualitative). Over half of the 25 studies identified for inclusion were conducted within the last six years (2010 or later). Most of these studies originated in Europe (*n* = 14), some in Australia or New Zealand (*n* = 7), and Asia (*n* = 3) and one in the USA. Most studies were set in the community, only one study included nursing-home residents. Fifteen studies included participants who had actually started some form of fall prevention exercises while nine studies did not encompass an actual intervention but investigated older adults’ intention to undertake fall prevention interventions, including exercise. One study included people with experiences of participation and non-participation. The type of exercises involved in the studies were group based (*n* = 6), home based (*n* = 4) and a mix of both home and group exercises (*n* = 5). The exercise programs generally included both strength and balance components, including The Otago Exercise Programme [48] and Falls Management Exercise - FaME [49], one study investigated Tai Chi [29] and one study aqua-aerobics [35].

**Table 1:** Summary of aim, methods, participants and reported results on gender analysis, if applicable, for the included quantitative studies and studies with a mixed design

Article	Aim	Methods	Participants	Reported gender analysis
Whitehead et al. [43]	Investigating the reason for not taking up fall or injury prevention strategy among older people who have sustained a fall and attended an emergency department.	Structural individual interviews Results presented in counts and percentages	60 (44 women and 16 men) community dwelling participants who attended the emergency department of a public hospital with a fall. Mean age 78 years. Country: Australia	Considerably more women than men mentioned already being active enough (women 27.3%, men 18.8%); can’t do exercises (women 22.7%, men 12.5%); and can’t leave house/spouse (women 13.3%, men 0%), as reason for reluctance to take part in exercise classes.
Yardley et al. [46]	To determine whether threat or coping appraisal are most closely related to older people’s intention to undertake strength and balance training.	Postal survey (n. 451) and structural interviews (n.107) Structural equation modeling	558 (397 women and 161 men) older people. Aged between 60 and 95 years, mean age 74. Country: UK	Younger respondents and women were somewhat more positive in their coping appraisal than were older respondents and men. Female gender was positively related to threat appraisal (*r* = .11, *p* = 009) and coping appraisal (*r* = .18, *p* = .001). Women were slightly less inclined than men to undertake SBT.
Yardley et al. [47]	To determine the extent to which older people, in different sectors, are willing to engage in different falls prevention activities.	Postal survey Logistic regressions	5440 (2846 women and 2482 men) patients from 10 general practices. Aged > 54 years. Country: UK	Substantially more women than men indicated that they were likely to attend group sessions (*p* < 0.001), and carry out SBT at home (*p* < 0.001).
Lin et al. [32]	To explore attitudes and beliefs of Taiwanese older women regarding SBT programs and their intentions to attend such programs.	Survey Multiple linear regressions and Pearson’s correlations	221 women recruited from college for people with a wish to learn in later life. Aged between 55 and 94 years, mean age 72. Country: Taiwan	Only women included.
Snodgrass and Rivett [39]	To explore the views and perceptions of older people about falls and falls injury prevention services, to identify incentives and barriers to attending a falls injury prevention service.	Survey 95% confidence interval (CI)	75 members of community groups. No experience of fall prevention exercises required. Aged between 61 and 93 years, mean age 74. Sex not reported. Country: Australia	Statistics not reported according to gender.
Hedley et al. [26]	To explore the reasons why the participants either did or did not adhere to an RCT intervention with both group and home exercises.	Mixed design: Qualitative: Individual interviews and one focus group Quantitative: Attendance rates, gait and balance assessments Thematic analyses and descriptive statistics	5 community dwelling women. Participants in the Staying Steady program with 32 weeks of group and home based exercises. Aged between 60 and 88 years, mean age 77. Country: UK	Only women included.
Robinson et al. [36]	To explore the process of behavior change in a small sample of older people with the fall-associated chronic liver disease primary biliary cirrhosis (PBC) receiving either a standard or an enhanced program of strength and balance training.	Mixed design: Individual interviews and graphical representations of patient-reported outcomes measures (PBC-40; FES-I; SEE Scale) Critical realist paradigm of enquiry	9 community dwelling women with PBC who participated in a 6-week or 6-month strength and balance training program. Aged between 63 and 80 year. mean age 70 year. Country: UK	Only women included.

**Table 2:** Summary of aim, methods, participants and reported results on gender analysis, if applicable, for the included qualitative studies

Article	Aim	Methods	Participants	Reported gender analysis
Clark et al. [24]	To explore older, community-dwelling adults’ attitudes and values about proposed church-delivered balance classes for fall prevention to inform a social marketing campaign.	Focus groups (*n* = 6) Inductive analysis	60 church members and potential users of the fall prevention exercise classes. Aged ≥60 year. Sex not reported. Country: USA	Women respond to a fall-prevention message more than men, and men rely on women for motivation. Women’s gendered identities positioned them to be the primary motivators who could soften their “stubborn” men to enroll in health programs. Men’s gendered identities positioned them to protectively identify women as high-priority recipients of balance and fall-prevention messages.
Jagnoor et al. [30]	To investigate fall prevention as a health priority among older people; to understand people’s perception of risk and concepts of fall injury prevention; and to explore acceptability of yoga as an intervention for falls prevention in the community.	Focus groups (*n* = 6) Thematic analysis	Gender divided focus groups in three sociodemographic clusters. 12–18 participants in each constellation. Aged >60 year. Sex not reported. Country: India	The experience, knowledge, perceptions and health priorities were diverse and differed across the three sociodemographic groups, although these were similar among men and women within each sociodemographic group. Women considered themselves active enough with domestic work. No gender discussion.
Berlin Hallrup et al. [23]	To explore the lived experience of fall risk from a life world perspective.	Individual interviews Phenomenological reflective lifeworld approach	13 community dwelling older women with previous fragility fractures. Participants in a hip fracture prevention program comprising a bone mineral density scanning, and written fall preventive advice including advice on exercises. Aged between 76 and 86 years. Country: Sweden	Only women included.
Hawley [25]	To explore what might encourage older people to exercise at home after falls rehabilitation.	Individual interviews Grounded theory approach	8 community dwelling, 1 nursing home resident. All had been through falls rehabilitation and offered home exercise programs Aged ≥60 year. Sex not reported. Country: UK	Quotes from both men and women but no comparison.
Horne et al. [27]	To explore the beliefs of both South Asian and White British community dwelling older adults in their 60s about falls and exercise for fall prevention.	Focus groups (*n* = 15) and individual interviews Framework analysis	87 + 40 (81 women and 46 men) community dwelling participants. Aged between 60 and 70 year, Focus group mean age 65. Individual interviews mean age 64. Participants with different experiences of participation or nonparticipation in exercise. Country: UK	Quotes from both men and women but no comparison.
Horne et al. [28]	To identify salient beliefs that influence uptake and adherence to exercise for fall prevention among community-dwelling Caucasian and South Asian in UK.	Ethnographic study participant observations, focus groups (*n* = 15) and individual interviews Framework analysis	87 + 40 (81 women and 46 men) community dwelling participants. Aged between 60 and 70 year, Focus group mean age 65. Individual interviews mean age 64. Participants with different experiences of participation or nonparticipation in exercise. Country: UK	Quotes from both men and women but no comparison.
Hutton et al. [29]	To develop an understanding of the perceptions that older adults at risk at of falls, and previously involved in organized group exercise, have of physical activity.	Focus groups (*n* = 5) Thematic analysis	20 community dwelling persons (90% females) recruited from a RCT investigating the effectiveness of Tai Chi in reducing falls. Aged between 68 and 81 year, mean age 73. Country: New Zealand	Gender not reported in quotes.
Lam et al. [31]	To examine older people’s perceptions and experiences of falls, physiotherapy and exercise.	Individual interviews Phenomenological approach	19 (10 women and 9 men) community dwelling Australian-born and Italian-born older persons who had more than one fall in the past 12 months and completed a community-based physiotherapy program. Aged between 65 and 89 years. Country: Australia	Quotes from both men and women but no comparison.
Lindgren De Groot and Fagerström [33]	To describe motivating factors and barriers for older adults to adhere to group exercise in the local community.	Individual interviews Descriptive content analysis	10 (5 women and 5 men) community dwelling persons and former participants in a fall preventive exercise program. Aged between 71 and 91 year, mean age 83. Country: Norway	Gender not reported in quotes.
Meyer et al. [34]	To understand the perspectives of older people in adopting a home-based balance exercise program to address mild balance dysfunction, and to identify barriers and opportunities facing community health PTs in delivering this program.	Focus groups (*n* = 2) with older participants, (focus groups, written surveys, and data recording sheets for PTs). Phenomenological approach. Thematic content analysis	9 (6 women and 3 men) community dwelling participants who had completed a six-month program. Aged between 67 and 86 years. 10 PTs Country: Australia	Gender not reported in quotes
Moody et al. [35]	To investigate participants’ perceptions of a twelve week aqua-aerobics program on falls risk and physical function in older adults with lower extremity osteoarthritis.	Focus groups (*n* = 4), one individual interview General inductive approach	17 (13 women and 4 men) community dwelling participants with lower extremity osteoarthritis who had completed a 12 week water-based exercise program. Aged between 68 and 89 years, mean age 78. Country; New Zealand	Gender not reported in quotes.
Robinson et al. [37]	To involve older people and PTs in the development of acceptable strategies to promote uptake and adherence with an exercise-based fall prevention program	Focus groups (*n* = 3) with older people and with local PTs (*n* = 4) Framework analysis	12 (8 women and 4 men) older people attending a regional falls and syncope service including exercises. Aged between 72 and 88 years, mean age 79. 18 (14 women and 4 men) PTs in the region. Country: UK	Quotes from both men and women but no comparison.
Simpson et al. [38]	To examine the extent to which older people are willing to adopt any of the following strategies in order to avoid falling: balance and lower limb strengthening exercises. home safety advice, and ‘taking care’.	Individual interviews Method of analysis not reported	32 (26 women and 6 men) persons discharged from acute elderly care medical wards. No experience of fall prevention exercises required. Aged >65 years, mean age 83. Country: UK	Quotes from both men and women but no comparison.
Stathi and Simey [40]	To explore the exercise experiences of nursing home residents who participated in a 6-month falls prevention exercise intervention.	Individual interviews, 14 at baseline and 7 at follow-up Interpretive phenomenological analysis	14 (12 women and 2 men) nursing home residents who participated in a 6-month chair-based exercise program. Aged between 86 and 99 years. Country: UK	Quotes from both men and women but no comparison.
Suttanon et al. [41]	Identify factors that influence commencement and adherence to a home-based balance exercise program for older people with mild to moderate Alzheimer’s disease (AD).	Individual interviews Phenomenological theoretical framework	10 (7 women and 3 men) participants with AD and 9 (6 women and 3 men) of their caregivers, who had completed a six-month home-based balance exercise program. Participants with AD aged between 75 and 89 years, mean age 81. Caregivers aged between 58 and 85 years, mean age 71. Country: Australia	Mostly quotes from women, no comparison.
Vernon and Ross [42]	To explore the reasons older people had for attending local postural stability exercise classes.	Individual interviews, 22 at baseline and 17 at follow up Thematic analysis	22 (20 women and 2 men) community dwelling participants who had fallen and attended balance exercise classes. Aged between 65 and 94 years. Country: UK	Gender not reported in quotes.
Wong et al. [44]	To estimate the uptake rate of a fall prevention program To explore the attitudes towards acceptance of the exercise class included in a fall prevention program. among older fallers and explore related factors	Focus groups (*n* = 3) Content analysis (Baseline telephone interviews (n. 1194) and a 1-year follow-up telephone survey (n. 969) with older people or their carers)	Focus Groups: (9 women and 4 men) previous fallers among who attended exercise classes based on the FaME protocol. Aged between 65 and 91 year, mean age 76. Country: Hong Kong	Gender not reported in quotes.
Yardley et al. [45]	To identify factors common to a variety of populations and settings that may promote or inhibit uptake and adherence to falls related interventions	Individual interviews Framework analysis	69 (50 women and 19 men) older people, two thirds (46) had been offered an intervention, and half (32) had taken part in an intervention. Aged between 68 and 97 years, mean age 79. Countries: Denmark, Germany, Greece, Switzerland, The Netherlands, and UK	Quotes from both men and women but no comparison.

The most common data collection method used to explore participant’s views and preferences of fall prevention exercises was one to one interviews (*n* = 13) followed by focus group discussions (*n* = 10) and surveys (*n* = 4). Several studies used a mix of qualitative methods. Mixed methods with both qualitative and quantitative design was used in two studies.

The number of participants in all included studies was about 7000. About 90% of the participants were included in studies involving postal surveys. All studies, except two [32, 47], had a minimum age of 60 years for inclusion and, based on available information, the mean age of all participants was approximately 76 years. Three of the studies did not report the gender distribution of the sample [24, 30, 39] but in the remaining studies the mean proportion of included women was 76%.

Not only were participants primarily female, but the authors were as well. The first author was a female in 88% of the studies and the mean proportion of male authors was 20%. This may be a reflection of the disciplines of the authors. The first author was a physiotherapist in 40% and a nurse in 28% of the studies each. The remaining studies were authored either by medical doctors, health psychologists, occupational therapists or other experts in public health.

### Gendered views of older adults to falls prevention exercise

Only five of the 25 articles retrieved in this review included some sort of gender analysis, regarding similarities or differences in men’s and women’s perceptions on fall prevention exercises [24, 30, 43, 46, 47]. None of these studies included participants already taking part in exercise to prevent falls. Two of these gender analyses were based on surveys, two on focus group discussions and one on interviews. In the articles without an explicit gender analysis (*n* = 20) the sex of the quoted participants was reported in 56% of the qualitative papers. Four studies included women only.

Results from the limited gender analysis performed indicate that men protectively identify women as high-priority recipients of balance and fall prevention exercise and that women see themselves as more receptive to fall prevention messages than men [24]. In addition the results indicate that men rely on women for motivation to enroll in health programs [24]. Even though many women seem to consider themselves as already active enough in their everyday life [30, 43], significantly more women than men are likely to attend group sessions [47]. However, the results seem inconclusive as to whether women are more or less inclined than men to undertake strength and balance training specifically [46, 47] (Table 1). No studies contained information on women and men’s specific preferences for program characteristics (e.g. approach) in exercise to prevent falls. See summary in Tables 1 and 2.

### Older participants views and preferences on fall prevention exercise

Facilitators and barriers for taking part in fall prevention exercises, expressed by older women and men as a group, from the 25 included studies, are reported in Table 3. The three most commonly occurring themes emerging as facilitators for commencement or adherence to an exercise program were: *support from professionals or family*; *social interaction;* and *perceived benefits*. In addition to these frequently represented themes, three other themes emerged: *a supportive exercise context; feelings of commitment*; and *having fun*. Being recommended or invited to join fall prevention exercise by a professional or having a professional instructor giving individually tailored exercises were repeatedly reported as important for improving uptake and adherence, as was social support and approval from family and friends. Social interaction was a recurrent theme in the studies as an important factor of relevance for adherence, it could sometimes even be a goal in itself and thereby important for uptake as well. Another theme frequently occurring in the studies was the importance of perceived benefits, meaning that the exercises were recognized as beneficial for improving or maintaining personal health and in particular for staying independent. The importance of a supportive exercise context was found in several studies, and included aspects such as an atmosphere of trust, small classes and easy access to exercise venues. Feelings of commitment was another recurrent theme reflecting the participants’ willingness to pursue a program (adherence) and to do it well, but also to contribute, for example by reducing a caregivers burden or contribute to research. The last theme, having fun, emphasizes the importance of enjoyment and playfulness as incentives for both uptake and adherence.

**Table 3:** Summary of the thematic analysis of facilitators and barriers reported in all studies. Views and preferences reported were coded and similar codes grouped into themes

Examples of codes	Themes	Reported in quantitative or mixed studies (see Table 1)	Reported in qualitative studies (see Table 2)
Facilitators			
“Recommendations from health professionals” “Support from professional” “Professional exercise instructor” “Family support” “Social approval “ “Positive social identity” “Being accompanied by a friend”	Support from professionals or family	Quantitative: Yardley et al. [46], Snodgrass and Rivett [39], Lin et al. [32] Mixed: Hedley et al. [26], Robinson et al. [36]	Hawley [25], Hutton et al. [29], Moody et al. [35], Stathi and Simey [40], Suttanon et al. [41], Wong et al. [44], Yardley et al. [45], Meyer et al. [34]
“Socialization” “Relationships” “Valued companionship” “Enjoyable and sociable atmosphere” “Having a coffee/tea as part of activity” “prefer group exercises”	Social interaction	Quantitative: Snodgrass and Rivett [39] Mixed: Hedley et al. [26]	Clark et al. [24], Hawley [25], Hutton et al. [29], Jagnoor et al. [30], Lindgren De Groot and Fagerström [33], Moody et al. [35], Stathi and Simey [40], Vernon and Ross [42]
“Staying independent” “Increase independence” “Functional improvements” “Maintaining health” “Believe that exercise has benefits” “Feeling improvements” “Improved mental health” “Reduction of fall risk” “Recent falls” “improved self-efficacy”	Perceived benefits	Quantitative: Yardley et al. [46, 47], Lin et al. [32] Mixed: Hedley et al. [26], Robinson et al. [36]	Clark et al. [24], Hawley [25], Jagnoor et al. [30], Lam et al. [31], Lindgren De Groot and Fagerström [33], Meyer et al. [34], Moody et al. [35], Simpson et al. [38], Stathi and Simey [40], Suttanon et al. [41], Vernon and Ross [42], Yardley et al. [45]
“Trust-based atmosphere” “Small size classes” “Suitable and nearby facility” “At home or group” “Participants of similar age” “Program characteristics” “Individually adapted” “Feeling ownership of the program” “High self-efficacy”	A supportive exercise context	Quantitative: Lin et al. [32], Yardley et al. [47] Mixed: Robinson et al. [36]	Berlin Hallrup et al. [23], Horne et al. [27], Hutton et al. [29], Lam et al. [31], Meyer et al. [34], Robinson et al. [37] Suttanon et al. [41], Vernon and Ross [42], Wong et al. [44]
“Commitment to a structured program” “Exercise recording sheet” “Measurable goals” “Minimizing caregivers burden” “Contribute to research”	Feelings of commitment		Meyer et al. [34], Moody et al. [35], Stathi and Simey [40] Suttanon et al. [41]
“Interest and enjoyment” “Enjoyable and joyful” “Activity sounds like fun”	Having fun	Quantitative: Snodgrass and Rivett [39]	Berlin Hallrup et al.[23], Suttanon et al. [41], Yardley et al. [45]
Barriers			
”Transportation to exercise venue” “Environmental factors” “Lack of suitable place at home” “Lack of time” “Bad weather”	Practical issues	Quantitative: Snodgrass and Rivett [39], Whitehead et al. [43] Mixed: Hedley et al. [26]	Horne et al. [27, 28], Hutton et al. [29], Lindgren De Groot and Fagerström [33], Moody et al. [35], Suttanon et al. [41], Vernon and Ross [42], Wong et al. [44], Yardley et al. [45]
“Fear of adverse effects” “fear of falling again” “Anxiety at start” “Unable to keep up with others in class” “A competitive atmosphere” “Too difficult exercises” “Different functional levels among participants” “Previous unpleasant experiences” “Dislike group activities” “Program not tailored”	Concerns about exercise	Quantitative: Snodgrass and Rivett [39] Yardley et al. [46] Mixed: Robinson et al. [36]	Horne et al. [27, 28], Hutton et al. [29], Lam [31], Lindgren De Groot and Fagerström [33], Moody et al. [35], Simpson et al. [38], Stathi and Simey [40], Suttanon et al. [41], Vernon and Ross [42]
“Misunderstandings of benefits” “Denial of risk of falling” “Perceive oneself as too young and fit” “Being active enough”	Unawareness	Quantitative: Snodgrass and Rivett [39], Whitehead et al. [43]	Horne et al. [27, 28], Jagnoor et al. [30], Simpson et al. [38] Yardley et al. [45]
“Deterioration in health” “Pain and pathology” “Feeling unwell” “Fatigue” “Feeling too old”	Reduced health status	Quantitative: Whitehead et al. [43]	Hutton et al. [29], Lindgren De Groot and Fagerström [33], Moody et al. [35], Simpson et al. [38], Suttanon et al. [41], Vernon and Ross [42]
“Unprofessional instructor” “Withdrawal of professional support” “Lack of support from home” “Caregivers health”	Lack of support	Quantitative: Whitehead et al. [43] Mixed: Hedley et al. [26]	Hutton et al. [29], Stathi and Simey [40], Suttanon et al. [41], Wong et al. [44]
“Lack of motivation” “Not interested”	Lack of interest	Quantitative: Snodgrass and Rivett [39], :Whitehead et al. [43]	Hutton et al. [29], Lindgren De Groot and Fagerström [33]

The barriers for taking part in fall prevention exercise were expressed in six themes: *practical issues; concerns about exercise; unawareness; reduced health status; lack of support*; and *lack of interest*. Practical issues were mentioned in the majority of the studies as the reason for not taking part in exercises. Such barriers were often related to transportation to exercise venues, but could also be lack of time, bad weather, or lack of a suitable place to exercise at home. Another barrier that was highlighted in many studies was the concerns older people had about the exercise. Many older people voiced a lack of confidence to do exercises and fear of adverse effects or a new fall. Concerns about not being able to ‘keep up’ with demanding exercises, or what other participants could manage, were also expressed. At the same time, for those who were functionally able, groups composed of participants at lower functional levels could also be frustrating. Unpleasant experiences from previous exercise or dislike of group exercises with a competitive atmosphere could also pose a barrier for taking part. Unawareness was another barrier and included lack of knowledge about specific benefits of exercise for fall prevention, older people perceiving they were already active enough, and denial of fall risk. Reduced health, such as pain, fatigue or other illness, as well as lack of support in their situation was another barrier. Some older people simply expressed that they were not interested in fall prevention exercises.

It was not possible to determine whether older adults preferred group or home based exercises as studies rarely offer both approaches. It seemed that the type of intervention included in the particular studies affected the participants’ preferences, so that they preferred what they had been offered. The results of the few studies offering both alternatives suggest that home exercises are more difficult to adhere to than the group sessions [26] and that older people rely on their own judgment when deciding on whether to take part in group or individual exercise, based on their exercise abilities, transportation issues, and willingness to expose their disabilities to others [23].

## Discussion

The main aim of this review was to explore any gender differences in views about or preferences for falls prevention exercise within the current literature. Unfortunately, only five of the articles in this review included a gender analysis of similarities or differences in men’s and women’s perceptions on fall prevention exercises, and none of these investigated if specific approaches attracted women or men differently. Despite the difficulties in making conclusions due to the differing designs and small number of studies, in terms of ‘views’, the results suggest that women are seen as high-priority recipients of balance and fall prevention messages by both women and men. Though incidentally, perhaps researchers also view women as high-priority participants as the mean proportion of included women in the studies was 76%. Women seem more receptive to fall prevention messages compared to men and are more likely to attend group sessions. No studies considered preferences of men and women and whether these differed or would affect uptake or adherence.

If services want to attract both older women and men to fall prevention exercise they need to consider how these programs are marketed and designed, taking into account the preferences of older men and women. A large consumer market research study based on numerous focus group discussions, individual interviews and surveys concluded that messages must move beyond transmission of basic health information and focus on encouragement and inspiration, while being careful not to alienate [50]. Messages will have more meaning if they are informed by knowledge on potential gender specific preferences.

With respect to the gender, according to this review it seems as if men are often not considered as being in need of fall prevention, both by themselves and society in general. These results conform to recent research showing that men to a lesser degree report and discuss falls and fall prevention with a healthcare provider [15]. One large survey to gain insight into the barriers to recruiting and engaging older men in evidence-based health promotion programs found that 78% of the respondents agreed that the perception of exercise programs as feminine was a barrier and over 90% of the survey respondents believed program advertisements featuring men would increase their participation [51].

The reason for this pattern can be discussed in relation to constructions of masculinity and gendered identities. From this perspective, gender does not reside in the person but is viewed as a dynamic, social structure in which men and women conforms to stereotypic beliefs and behaviors based on dominant norms of femininity and masculinity. According to the norms, men should be independent, self-reliant, strong, tough and willing to take risks [52]. In addition, this view of masculinity includes denial of weakness or vulnerability and rejection of feminine ideals, which include positive health beliefs or behaviors. These gendered identities are believed to foster both unhealthy behavior among men and undermine men’s attempts to adopt healthier habits [53]. It is important to know if men are less likely to take up falls prevention exercise as this could be considered as them being frail and needing help to maintain independence. For example, one study gave a quote from a Greek man “*You should be very careful about the way you would approach old men and tell them that they might need to participate in this…. Not everybody accepts his age and his state*” [45]. Would joining a group program of exercise with women be a threat to their identity? These are questions we cannot answer with the current literature. Of course, it is also possible that exercise preferences for older men and women with a history of falls may not differ, as is the case with men and women with multiple sclerosis [54]. However, with the much smaller number of men included in studies on exercise to prevent falls we cannot be sure that part of the reason is the lack of identification with such programs by men.

A clear trend when investigating participant’s preferences for fall prevention exercise, was that women do research on and for women. If researchers or clinicians tend to favor the inclusion of women in prevention programs, gender bias (conscious or unconscious) may arise and stereotype gendered views and identities may be confirmed [55]. Researchers and clinicians need to be aware of their own gendered identities and how they are influenced by gender relations [56] in order not to strengthen or reinforce stereotypical gender views.

This review has, from 25 included studies, identified six themes as *facilitators* and six themes as *barriers* for older people either starting or adhering to fall prevention exercise, irrespective of gender (Table 3). The identified facilitators and barriers concur well with the results of previous (non-gender specific) reviews, which included perceptions of fall prevention interventions including exercise [13, 14]. Recurrent themes were the need for social and professional support and that exercise is perceived as beneficial and important for maintaining independence. Barriers related to practical issues, like access to programs and lack of support, were consistent [14] as were perceived poor health, poor self-efficacy, fear of falling, underestimation of fall risk and concerns about exercise [13].

The psychosocial factors expressed by the older participants as important for engagement in fall prevention exercises conforms well with many health psychology theories commonly used to predict health behaviors, in particular the theory of planned behavior (TPB) [57]. This theory has previously been applied in fall prevention research [58] and was also used in four of the included studies [27, 32, 45, 46]. From the 25 studies in this review it is apparent that older people’s attitudes to the effects of exercises are mainly positive. Older people are often aware of the beneficial effects of physical exercise to improve their general health, and this knowledge motivates them to be active. However, exercise performed merely with the goal to prevent future falls, does not seem enough to attract this population. This attitude has previously been explained by two coupled factors: (a) a belief that falls cannot be prevented, but are caused by external circumstances and bad luck, and (b) an underestimation of the personal perceived fall risk [58]. The influence of a subjective norm was confirmed as essential for many older people’s commencement or adherence to an exercise program. Both professional and social support as well as social interaction during fall prevention exercise was commonly reported as important factors in the studies reviewed. Many of the barriers reported related to a perceived lack of control over the exercise situation, such as practical issues and various concerns about the exercises including lack of confidence, worries about not being able to keep up with others, and fear of adverse effects.

A number of methodological features could influence the result of this review. A strength is the broad literature search in a number of electronic databases, done systematically with the consultation of a librarian. However, despite the efforts to identify all eligible publications, we cannot exclude the possibility that some were missed. The lack of generally used keywords for participant’s views and preferences and the diverse research methodologies used in the studies aiming for made it difficult to develop a comprehensive search strategy. In order to compensate for this difficulty, the reference lists of all included articles and previous resembling reviews were searched for additional publications, which resulted in a few more studies to include. No ranking of quality of the included studies was performed.

## Conclusion

In conclusion, although there is plenty of information on the facilitators and barriers to falls prevention exercise in older people, there is a distinct lack of studies investigating differences or similarities in older womens’ and mens’ views regarding fall prevention exercise. In order to ensure that fall prevention exercise is appealing to both sexes and that the inclusion of both men and women are encouraged, more research is needed on preferences to find out whether any gender differences exists and whether and how practitioners need to offer a range of opportunities and support strategies to attract both women and men to falls prevention exercise.

## Additional file

Additional file 1: Literature search. (PDF 152 kb)
