# Supplementary material for: Gender perspectives on views and preferences of older people on exercise to prevent falls: a systematic mixed studies review
Source: BMC Geriatr. 2017 Feb 17;17:58. doi: 10.1186/s12877-017-0451-2 (PMC5316178; doi:10.1186/s12877-017-0451-2)
Supplement: Additional file 1: — Literature search. (PDF 152 kb) [file 12877_2017_451_MOESM1_ESM.pdf]

Search strategies and numbers of records identified in each database

Date of searches: 7th October 2013, 15th May 2014, and 8th February 2016

PubMed

1. MeSH "Accidental Falls"[Majr] Filters: Humans; English (7121 + 88 +777)
2. (falls OR faller* Filters: Humans; English (27393 + 264 + 2425)
3. 1 OR 2 (27393 + 264 + 2425)*
4. MeSH "Aged"[Mesh] Filters: Humans; English exploded (1757349 + 13265 +118968)
5. (older OR senior OR seniors OR elderly[Title/Abstract]) Filters: Humans; English (308029 + 2859 + 24019)
6. 4 OR 5 (1861681 + 14342 + 127031)
7. (experience* OR subjective OR perception* OR meaning OR meanings OR view OR views) [Title/Abstract]) Filters: Humans; English (813026 + 5461 +51641)
8. (exercis* OR "physical activity" OR strength* OR balance) [Title/Abstract]) Filters: Humans; English (369167 + 2975 +26918)
9. **3 AND 6 AND 7 AND 8 (402 + 3 + 57=462)**

CINAHL

Limits: peer review, humans , English

1. (MM "Accidental Falls") (2927 + 164 +483)
2. AB (falls or faller*) OR TI (falls or faller*) (4098 + 197 + 2998)
3. 1 OR 2 (4800 + 244 + 2998)
4. (MH "Aged+") (198 063 + 7953 + 25 145)
5. AB (older OR senior OR seniors OR elderly) OR TI (older OR senior OR seniors OR elderly) (59 843 + 2499 + 7115)
6. 4 OR 5 (215 021 + 8621 + 26 978)
7. AB (experience* OR subjective OR perception* OR meaning OR meanings OR view OR views) OR TI (experience* OR subjective OR perception* OR meaning OR meanings OR view OR views) (121 282 + 5285 + 13 702)
8. AB (exercis* OR "physical activity" OR strength* OR balance) OR TI (exercis* OR "physical activity" OR strength* OR balance) (56 515 + 2658 + 7406)
9. **3 AND 6 AND 7 AND 8 (135 + 12 + 44=191)**

Amed

Limits: English

1. (DE "ACCIDENTAL FALLS") (1391 + 7 +77)
2. AB (falls or faller*) OR TI (falls or faller*) (1537 + 5 + 71)
3. 1 OR 2 (1933+ 7 + 83)
4. (DE "AGED") OR (DE "AGING") (1721 + 5 + 77)
5. AB (older OR senior OR seniors OR elderly) OR TI (older OR senior OR seniors OR elderly) (10 843 + 15 + 305)
6. 4 OR 5 (11 876 + 15 + 348)

7. AB (experience* OR subjective OR perception* OR meaning OR meanings OR view OR views) OR TI (experience* OR subjective OR perception* OR meaning OR meanings OR view OR views) (23 800 + 33 +526)
8. AB (exercis* OR “physical activity” OR strength* OR balance) OR TI (exercis* OR “physical activity” OR strength* OR balance) (23 657 + 50 + 691)
9. **3 AND 6 AND 7 AND 8 (62 + 0 + 4=66)**

PsycINFO

Limits: peer review, humans , English,

1. DE "Falls" (1235 + 57 + 305)
2. AB (falls or faller*) OR TI (falls or faller*) (12 337 + 346 + 1743)

1 OR 2 (12 393 + 348 +1763)

4. DE "Aging" (22 904 + 963 + 4832)
5. AB (older OR senior OR seniors OR elderly) OR TI (older OR senior OR seniors OR elderly) (105 052 + 2485 + 12 483)
6. 4 OR 5 (110 802) (*with limiter Age Groups: Aged (65 yrs & older), Very Old (85 yrs & older) (58 055 + 1518 + 13 531)*
7. AB (experience* OR subjective OR perception* OR meaning OR meanings OR view OR views) OR TI (experience* OR subjective OR perception* OR meaning OR meanings OR view OR views) (448 251 + 11 425 + 52 867)
8. AB (exercis* OR “physical activity” OR strength* OR balance) OR TI (exercis* OR “physical activity” OR strength* OR balance) (93 613 + 3093 + 14 934)
9. **3 AND 6 AND 7 AND 8 (73 + 4 + 24=101)**

Scopus

TITLE-ABS-KEY((falls OR faller*) AND (older OR senior OR seniors OR elderly) AND (experience* OR subjective OR perception* OR meaning OR meanings OR view OR views) AND (exercis* OR "physical activity" OR strength* OR balance))

Limits: (LIMIT-TO(SUBJAREA, "MEDI") OR LIMIT-TO(SUBJAREA, "HEAL") OR LIMIT-TO(SUBJAREA, "NURS") OR LIMIT-TO(SUBJAREA, "SOCI") OR LIMIT-TO(SUBJAREA, "PSYC")) AND (LIMIT-TO(DOCTYPE, "ar") OR LIMIT-TO(DOCTYPE, "re")) AND (LIMIT-TO(EXACTKEYWORD, "Human") OR LIMIT-TO(EXACTKEYWORD, "Aged") OR LIMIT-TO(EXACTKEYWORD, "Accidental Falls") OR LIMIT-TO(EXACTKEYWORD, "Aged 80 and over") OR LIMIT-TO(EXACTKEYWORD, "Aging") OR LIMIT-TO(EXACTKEYWORD, "Exercise") OR LIMIT-TO(EXACTKEYWORD, "Falls") OR LIMIT-TO(EXACTKEYWORD, "Physical activity") OR LIMIT-TO(EXACTKEYWORD, "Elderly care") OR LIMIT-TO(EXACTKEYWORD, "Balance")) AND (LIMIT-TO(LANGUAGE, "English")) **(488 + 19+112=619)**

PEDro

1. fall (915)
2. AND aged (217)
3. **AND Experience (13 + 1 + 1=15)**

OTseeker

(aged OR older OR senior OR seniors OR elderly) AND (falls OR faller OR fallers) AND (experience OR experiences OR subjective OR perception OR perceptions OR meaning OR meanings OR view OR views) **(16 + 0=16)** *(Database does not work properly in february 2016)*
